# Bio-psychosocial determinants of cardiovascular disease in a rural population on Crete, Greece: formulating a hypothesis and designing the SPILI-III study

**DOI:** 10.1186/1756-0500-3-258

**Published:** 2010-10-11

**Authors:** Christos Lionis, Dimitrios Anyfantakis, Emmanouil K Symvoulakis, Sue Shea, Demosthenes Panagiotakos, Elias Castanas

**Affiliations:** 1 Clinic of Social and Family Medicine, Faculty of Medicine, University of Crete, Greece; 2 School of Health and Social Care, University of Greenwich, UK; 3 Department of Nutrition-Dietetics, Harokopio University, Athens, Greece; 4 Laboratory of Experimental Endocrinology, School of Medicine, University of Crete, Heraklion, Greece

## Abstract

**Background:**

In 1988, the SPILI project was established in order to evaluate the cardiovascular disease (CVD) risk profile of the inhabitants of Spili, in rural Crete, Greece. The first reports from this project revealed that against the unfavourable risk factors' profile observed, only a few men with a previous myocardial infarction were encountered. A follow-up study (SPILI II) was performed twelve years after the initial examination, and the unfavourable cardiovascular risk profile was re-confirmed.

**Presentation of the Hypothesis:**

This paper presents a hypothesis formulated on the basis of previous research to investigate if dynamic psycho-social determinants, including social coherence of the local community, religiosity and spirituality, are protective against the development of coronary heart disease in a well-defined population.

**Testing the Hypothesis:**

A follow-up examination of this Cretan cohort is currently being performed to assess the link between psychosocial factors and CVD. Psychosocial factors including sense of control, religiosity and spirituality are assessed in together with conventional CVD risk factors. Smoking and alcohol consumption, as well as dietary habits and activity levels are recorded. Oxidative stress and inflammatory markers, as well as ultrasound measurement of carotid intima media thickness, a preclinical marker of atherosclerosis, will also be measured.

**Implications of the hypothesis tested:**

The issue of the cardio-protective effect of psycho-social factors would be revisited based on the results of this Cretan cohort; nevertheless, further research is needed across different sub-populations in order to establish a definite relationship. A comprehensive approach based on the aspects of bio-social life may result in more accurate CVD risk management.

## Background

Cardiovascular disease (CVD) represents the leading cause of mortality in both the industrialised and developing world [1]. Major risk factors on the pathogenesis and precipitation of CVD include age, gender, hypertension, dyslipidaemia, smoking and diabetes [2]. However, particular emphasis has also been given to the role of psycho-social determinants, such as social isolation, chronic life stress, anxiety, hostility and depression [3]. It has been reported that the etiologic link between psycho-social determinants and atherosclerosis may be through the maintenance of aggravating lifestyle behaviours and the discouragement of their modification, or due to direct endothelium damaging [3]. Evidence suggests that low socio-economic status, social isolation, lack of social support, familial and occupational stress, negative emotions including depression and hostility, may aggravate the prognosis and clinical course in patients with coronary heart disease (CHD) [4]. Data retrieved from human and animal studies link sympathetic nervous system hyperactivity, triggered by psychological stimuli, with accelerated development of carotid atherosclerosis [3]. It has been reported that chronic stress conditions and negative emotional states promote atherosclerosis through an increased output from the sympathetic nervous system and hypothalamic-pituitar-adrenal axis, leading to a variety of adverse peripheral effects, including inflammation [5]. It is also remarkable that psycho-social variables tend to synergistically interplay with conventional risk factors, enhancing the risk for cardiac events [3].

In 1988, a research project was launched, aiming to explore the cardiovascular risk profile of the inhabitants of Spili in rural Crete, Greece [6]. The study (SPILI I) was performed in a primary health care centre located in the Cretan village of Spili, and comprised of permanent residents (n = 445) aged 15-79 years. The overall attendance rate was 77% (n = 343). Despite an unfavourable profile in terms of smoking prevalence, hypertension, diabetes and increased alcohol intake, the study's investigators found only 1% (3) of the examined subjects with a previous myocardial infarction [6]. The investigators suggested a possible cardio-protective role related to the closely-knit social relationships, the low unemployment rate, and the potential benefit of certain dietary habits, such as the high consumption of olive oil [6].

A follow-up study was performed twelve years after the initial examination (i.e., in 2000), with the aim of describing the trends of CHD risk factors over time and discussing some key points on the natural course of the disease (SPILI II) [7]. The target population consisted of all inhabitants of Spili who were originally examined in 1988, and who were still living in the area [n = 248]. A total number of 200 people were re-examined (overall participation rate 80.7%). Hypertension prevalence had increased in almost every age group, while obesity represented a more important problem compared to 1988, most likely as a result of sedentary life style [7]. Furthermore, an unfavourable trend was observed for diabetes, particularly in women. Moreover, an increase in the number of middle-aged women (i.e., 45-64 years) who currently smoked was recorded. It is reported that Greece has the highest percentage of adult tobacco use worldwide [8]. Despite these findings, signs of clinically evident CHD were still scarce representing a challenging motivation for further research [7].

This article illustrates potential links between dynamic psycho-social determinants (social coherence of the local community, religiosity and spirituality) and coronary heart disease in a well-defined population sub-group on Crete with the aim to formulate a new Cretan hypothesis and discuss the design of a further ongoing follow-up study.

### Presentation of the hypothesis

Certain studies have attempted an explanation for the low CHD incidence on Crete, with a main focus on the Mediterranean diet [9]. Furthermore, specific life-style characteristics, such as adherence to the Greek orthodox religious fast, have been reported to exert a beneficial effect on lipidemic profile and prevalence of obesity [10]. Although interest in the link between psychosocial factors and CHD has increased in the literature in recent years, this subject still seems to be neglected in Greece [11]. Furthermore, the link between a positive psychosocial profile and CHD has not received the expected attention. Despite the high prevalence of smoking, alcohol intake, and a relatively high prevalence of hypertension, diabetes, obesity and hypercholesterolemia, the prevalence of CHD seems to be low in the rural population that we studied. This again raises questions concerning invisible factors contributing to the prevention of CHD. The presence of positive dynamic cardio-protective factors that counterbalance the influence of the previously reported CHD risk factors could offer a potential explanation. Common cultural and traditional aspects of the Cretan cohort, such as family and social support in the context of local communities and daily life management, may serve as a protective role against the manifestations of CHD.

In another study which was implemented in a remote area of Crete a plausible association between religiosity, spirituality and sense of coherence was discussed [12], offering additional thoughts in terms of the link between positive psychosocial factors and CHD in the SPILI project findings. We assume some patho-physiological mechanisms that explain the decreased cardiovascular risk in our studied population through a decreased chronic inflammation. In their review concerning psychosocial factors in the development of coronary artery disease, Strike *and *Streptoe discussed certain mechanisms through which social and psychological factors impact on coronary atherogenesis, and inflammation was among these [11]. In a UK study by Surtees P *et al*., a strong sense of coherence was associated with a 30% reduction in all-cause mortality among 20,500 participants, suggesting its possible protective role against the risk of chronic disease [13]. Further work supports that individuals with a greater sense of coherence present higher levels of self-esteem, optimism and control over their lives, making more likely a response to a stressor with adaptive mechanisms [14].

Therefore, we may hypothesize the existence of bio-psycho-social protective factors towards cardiovascular mortality and morbidity, among a Cretan population who is highly homogeneous in terms of ethnic and religious identity. Such factors could include, among others, religiosity and spirituality. The concurrent examination of psycho-social determinants in conjunction with biological markers in longitudinal studies is relatively rare [15]. Individuals who feel hopeless, unable to cope with stress or are socially isolated [3] are at considerable risk of developing CVD. More specifically, social determinants may be related to family status, friendships, social and religious group membership [16]. Thus, of particular importance to our project is the observation of a positive correlation between optimism and CVD focusing on sense of control. Given that the church is an important organization for socialization, and that religious beliefs and spirituality can have an impact on feelings of hopelessness or ability to cope with stress, leading to better outcomes [16], the above-cited elements carry great significance for the proposed study. It is also suggested that individuals with high levels of religiosity in terms of church attendance, religious activities and beliefs have better coping abilities, less depression and anxiety and decreased morbidity and mortality compared with those who are less religious [17]. Among explanatory mechanisms through which religious involvement leads to positive health outcomes seems to be the promotion of positive self perceptions and health beliefs, the regulation of individual lifestyles and health behaviours, and the provision of social ties and specific cognitive or behavioral response to stress [17]. Idler *et al. *suggest that people who are highly involved in worship activities have a greater sense of belonging to their faith group and more positive emotions, offering some novel pathways to be explored in order to better understand the linkage between health outcomes and religion [18].

With regard to the role of socio-economic status (SES) clinical practice guidelines of the European Society of Cardiology on cardiovascular disease prevention consider low SES as an aggravating parameter in the clinical course and prognosis of CHD [19]. Furthermore, it has been reported that other psychosocial risk factors such as depression, hostility, work and family stress tend to cluster among individuals and groups of low SES [19]. In alignment with this, a cross-sectional study among elderly people living on the Eastern Mediterranean islands found that subjects of higher SES were more close to the traditional Mediterranean diet as compared with those in the lowest SES [20]. Researchers suggested that factors such as educational status and income may exert a possible influence on the dietary habits of this population [20].

It is noticed that research regarding bio-psycho-social interactions and health has received limited attention in Greece. However, questionnaires and scales relevant to self-efficacy, sense of coherence [21] and spirituality [22] have been adapted and validated in Greek and are available for the measurement of unseen determinants [23,24]. Drawing on the above, we propose an interdisciplinary research effort, which will investigate the interaction between specific psycho-social determinants, religion and biological processes.

### Testing the hypothesis

**Figure 1:**
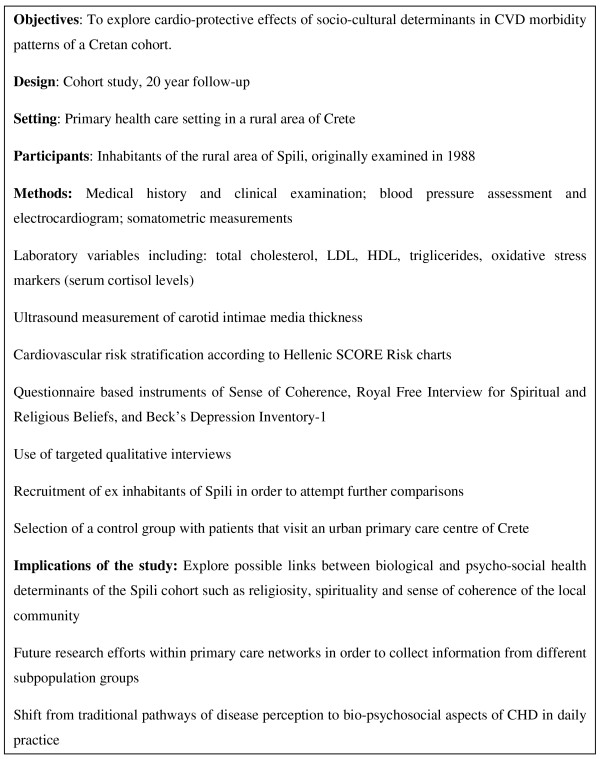
**Brief summary of the SPILI-III research protocol study**.

In order to confirm or refute the "Cretan hypothesis" outlined above, we will conduct a cohort study (SPILI III), which will evaluate the effect of cultural, religious and related psychosocial characteristics (shared within this rural population of Crete), with the relationship between traditional risk factors and CVD (Figure 1). Additionally, where possible, ex inhabitants of Spili who have moved to a more urban and stressful environment will also be recruited, to explore whether these individuals demonstrate a CVD pattern more comparable to global patterns. A control group with patients visiting an urban primary care centre in Crete will also be selected and will be matched with the Spili population group in terms of age and sex.

## Measures

### 1. Medical assessment

A complete medical history will be obtained from participants initially registered in the SPILI I study. Participants will undergo a blood pressure assessment, electrocardiogram and anthropometric measurements (height, weight, hip and waist circumference). Participants will be further evaluated for the presence of markers of atherosclerosis [i.e., fasting serum total cholesterol, low-density lipoprotein (LDL), high-density lipoprotein (HDL) triglicerides], markers of diabetes (i.e., fasting glucose), markers of inflammation [i.e., high sensitivity C-reactive protein (CRP), interleucin-6 (IL-6), fibrinogen] [25,26], as well as oxidative stress [27] and total antioxidant capacity [28]. Ultrasound measurement of carotid intima media thickness, a preclinical marker of atherosclerosis will also be performed [26,29]. The 10-year risk of fatal CVD will be calculated for all participants using the Hellenic SCORE Risk charts [4].

### 2. Behavioral assessment

Dietary patterns (MedDietScore) [30], activity levels (International Physical Activity Questionnaire) [31], smoking, and alcohol habits will also be recorded.

### 3. Psychosocial factors' assessment

Specific attention will be given to the social coherence of the local community [32] and their spiritual and religious beliefs and practices. Validated questionnaires will be used to evaluate sense of coherence (13 item Sense of Coherence Questionnaire) [21,33,34] religious and spiritual beliefs and practices (Royal Free Interview for Spiritual and Religious Beliefs) [22] and depression levels (Beck Depression Inventory-I) [35]. Socioeconomic background and poverty will be estimated after classification on the basis of the mean annual family income and years of education [20].

A further dimension will be added to the study by the performance of interviews with a sample of the cohort, which could provide additional insight into the qualitative findings. This could represent an opportunity to capture informants' understandings and explanations of their personal experiences of religiosity, spirituality and social coherence accessed within the context of a group defined by geography and common culture.

### Statistical analysis

Incidence rates of CHD and related risk factors will be calculated as the ratio of new cases developed during the preceding years to the number of person-years. Continuous variables will be presented as mean values ± standard deviation and categorical variables will be presented as frequencies. Associations between categorical variables will be tested using the chi-squared test. Comparisons of mean values of normally distributed variables between those who developed a CVD event and the rest of the participants will be performed using Student's t-test. For the continuous variables that were not normally distributed, the Mann-Whitney non-parametric test will be applied to evaluate the differences in the distributions of the skewed variables between those who developed a CVD event and the remaining participants. Cox PH models with first order interactions will be estimated to test the potential mediating effect of bio-social aspects on the effect of the traditional CVD risk factors on CVD incidence. Sample size calculation revealed that to assess a log{hazard ratio} equal to 0.08 (or hazard ratio = 1.20) with a standard deviation of 1.5, a sample of 550 cases is needed in order achieve 80% power at a 0.05 significance level (two sided hypotheses) [36].

### Ethical approval and confidentiality

Approval for this study was obtained by the Institutional Ethics Committee of the University Hospital of Crete (No Protocol:9989/02.09.2008). Written informed consent will be obtained from all participants. The anonymity of the patients' data will be carefully approached. The collected data will be stored at the Clinic of Social and Family Medicine, School of Medicine, University of Crete in which will be accountable from any public access.

### Implications of the hypothesis

The current proposed study aims to explore the extent to which Cretan individuals with greater sense of coherence and high religiosity and spirituality levels, are less likely to be affected by chronic inflammation and atherosclerosis related burden and abnormal levels of the relative bio-markers examined.

The findings will help researchers further explore possible links between biological and psychosocial health determinants. The study could also provide further evidence on the association of other factors such as the Mediterranean diet, which may explain a part of the CVD variation in the population studied. To achieve this, carefully designed research efforts are required in order to explore hypothetical patho-physiological pathways (direct and indirect) through which dynamic psycho-social determinants exert a protective biological effect and counterbalance a negative bio-profile. Furthermore, future interventions and health promotion programmes which take into consideration not only classical cardiovascular morbidity determinants (e.g. smoking, hyperlipidaemia and hypertension) but also adverse psycho-social risk factors may influence our understanding of CVD features in relation to its presentation and natural history. The proposed study may provide an explanation of the so-called "Cretan paradox", by interpreting a range of factors, which may have contributed to the low CVD incidence on the island of Crete during the last decades [37].

#### Recognized limitations

A weakness of the proposed study is the inevitably small population size and time related losses with regard to follow-up, since this group has already been monitored for almost two decades. Furthermore, we cannot easily reject or adopt scenarios such as the presence of other confounding factors due to genetic patterns within the study population and the beneficial effects of Christian Orthodox fasting on serum lipids and obesity. As Strike *and *Streptoe underline, it is often difficult to isolate the effects of independent psychosocial variables because they interact and tend to cluster together [11].

#### Concluding remarks

In this collaborative proposal, researchers intend to return to the Spili cohort and link biological, psycho-social and socio-cultural data into an overarching model that may provide a clearer understanding of how biological and psycho-social determinants of health intersect and impact on morbidity. The Spili cohort presents a rare opportunity to build on an already solid body of research by way of asking broader questions regarding the relevance of traditional practices and cultural heritage on morbidity and mortality at the individual and community level. We anticipate that certain patterns will be identified which could have both immediate applicability for current clinical and public health concerns, and which may also generate a new round of research questions with regard to the significance of the community for the health and well-being of the individual.

## Competing interests

The authors declare that they have no competing interests.

## Authors' contributions

CL conceived and shaped the idea. DA, EKS and CL prepared the first draft of the manuscript. DP provided intellectual input and information on future statistical analysis. SS corrected the manuscript and provided useful suggestions on content, together with editorial support. EC provided scientific and technical input. All authors read and approved the final manuscript.
